# Food insecurity among adolescent girls who are mothers (10–19 years) in sub-Saharan Africa: A scoping review

**DOI:** 10.1371/journal.pone.0346835

**Published:** 2026-09-30

**Authors:** Suliat Fehintola Akinwande, Kehinde Oluwatosin Akinwande, Tanzil Islam, Carmen Logie

**Affiliations:** 1 Factor-Inwetash Faculty of Social Work, University of Toronto, Toronto, Ontario, Canada; 2 Department of Oral and Maxillofacial Surgery, Lagos University Teaching Hospital, Lagos, Nigeria; 3 United Nations University Institute for Water, Environment, and Health, Hamilton, Ontario, Canada; 4 Women’s College Research Institute, Women’s College Hospital, Toronto, Ontario, Canada; Innovative Aid, CANADA

## Abstract

The population of adolescent girls in sub-Saharan Africa (SSA) is projected to reach approximately 160 million by 2030, with nearly one-third expected to transition into motherhood. Despite this, there persists a significant knowledge gap regarding the dynamics of food insecurity among adolescent mothers in a region that bears nearly 10% of the global burden of food insecurity. Marginalized adolescent mothers in resource-constrained settings experience heightened vulnerability to food insecurity due to structural disadvantages that reinforce intergenerational cycles of deprivation and impede progress toward achieving equity and improved well-being among adolescent mothers. We conducted a scoping review, following the Arksey and O’Malley framework enhanced by the Joanna Briggs Institute methodology and reported according to the Preferred Reporting Items for Systematic Reviews and Meta-Analyses extension for Scoping Reviews (PRISMA-ScR) guidelines, to examine the drivers, coping strategies, and mental, sexual, and reproductive health impacts associated with food insecurity among adolescent mothers (10–19 years) in SSA. We searched five databases and identified 263 non-duplicate records, of which 16 met the inclusion criteria. Thematic analysis revealed that the drivers of food insecurity were economic (poverty and economic constraints), social (stigma, family size, and caregiving responsibilities), and ecological (infertile land) factors. Coping strategies included economic-based activities, reliance on social support, and high-risk behaviours. Food insecurity had ripple effects on sexual and reproductive health, including coerced, age-disparate, and substance-influenced sex, and adverse mental health outcomes such as depression and suicide attempts. This review highlights the need for further research to drive meaningful advancements in policy and practice related to food security among adolescent mothers in SSA.

## Introduction

Adolescent motherhood is a significant global issue, with 21 million pregnancies occurring annually worldwide among girls aged 15–19, half of which are unintended [1]. Sub-Saharan Africa (SSA) reports the highest rate of childbirth among adolescent girls (aged 10–14) [1]. The health and social consequences of adolescent motherhood are particularly pronounced, as adolescence is a critical developmental stage marked by ongoing physical, emotional, and cognitive maturation [2]. This developmental vulnerability contributes to elevated maternal mortality and adverse social outcomes among adolescent mothers [3,4].

Prevailing social perceptions further compound the challenges of adolescent mothers, as they are often subjected to stigmatizing narratives that portray them as irresponsible or promiscuous [4]. Such stigma restricts access to education, healthcare, and social support, thereby deepening their exposure to resource insecurity, particularly food insecurity [5,6]. This is especially concerning in SSA, where hunger and food insecurity remain disproportionately high. Recent estimates indicate that more than 20% of the population in SSA experienced hunger in 2024, compared with 8.2% globally, and the region is projected to account for nearly 60% of the world’s chronically undernourished population by 2030 [7].

Food insecurity refers to limited or uncertain access to sufficient, safe, and nutritious food required for an active and healthy life [8]. Food insecurity ranges from low or moderate food insecurity, characterized primarily by compromised dietary quality and variety, to severe or very low food insecurity, in which food intake is reduced, and normal eating patterns are disrupted due to inadequate access to food [9]. It is important to distinguish food insecurity from hunger, which is a potential physiological consequence of more severe forms of food insecurity. Food insecurity is closely linked to a range of adverse psychosocial and physical health outcomes in adolescents, a population particularly vulnerable due to the rapid growth and development that characterizes this life stage [10]. These risks are amplified for adolescent girls who are pregnant or parenting. Compared to older mothers, adolescent mothers are more likely to be malnourished and to give birth to low-birth-weight infants, who themselves face heightened risks of malnutrition, illness, and mortality [11]. In addition to these physiological vulnerabilities, adolescent mothers frequently experience mental health distress driven by stigma, social exclusion, financial hardship, and unmet basic needs [12].

In SSA, where chronic hunger is prevalent, the convergence of adolescent motherhood and food insecurity may perpetuate a cycle of poor health outcomes [12–14]. Further, limited access to nutritious food, healthcare, and social support not only undermines the well-being of young mothers but also compromises the developmental trajectories of their children [11]. Addressing food insecurity is therefore essential to improving maternal and child health and mitigating the broader social consequences of adolescent motherhood. The food security of adolescent mothers and their children living in resource-constrained settings marked by intersecting poverty, hunger, and disease remains an urgent global concern.

Resource Scarcity Theory (RST) offers a multidimensional framework for understanding the drivers, coping mechanisms, and impacts of food insecurity [15]. RST conceptualizes shared social, economic, and ecological vulnerabilities to scarcity, coping strategies, and impacts on well-being [15]. It identifies five core predictors of scarcity: ecological conditions, population pressures, governance structures, market dynamics and entitlement systems that shape individuals’ legal and economic access to food. The coping strategies include modified consumption (e.g., reduced portions or stigmatized foods), migration, reprioritization of resources, and intensification of labour or asset liquidation [16]. RST highlights the physiological and psychological impact of resource insecurity on well-being, including stress, anxiety, and undernutrition. Food insecurity has also been linked to sexual health, including intimate partner violence (IPV) [17,18], transactional sex [19], and increased risk of recent HIV [18]. Applying RST to understand food insecurity not only illuminates the structural and psychosocial drivers of scarcity but also provides a foundation for identifying critical gaps in existing research.

Existing reviews have examined either the experiences of pregnant and parenting adolescents in Africa or the consequences of food insecurity among children and adolescents more broadly, but none has specifically focused on food insecurity among pregnant adolescents and adolescent mothers in SSA [20–22]. For example, Ajayi et al. (2023) synthesized the lived experiences of pregnant and parenting adolescents across Africa, highlighting challenges such as stigma, social exclusion, and barriers to healthcare and education. However, food insecurity was not the primary focus of the review, and its determinants, coping strategies, and health consequences were not comprehensively examined. Similarly, Kasujja et al. (2025) conducted a systematic review of food insecurity and mental health among children and adolescents aged 5–19 years in SSA and found significant associations between food insecurity and adverse mental health outcomes, including depression, mood problems, and other mental disorders. While this review provides important evidence on the impact of food insecurity among young people, it does not specifically address pregnant adolescents and adolescent mothers, a population that experiences unique vulnerabilities related to pregnancy, childcare responsibilities, gendered social expectations, stigma, and socioeconomic disadvantage [16,23].

To our knowledge, no previous review has synthesized evidence on food insecurity among pregnant adolescents and adolescent mothers in SSA. This scoping review addresses this gap by focusing specifically on this underserved population and mapping the existing evidence on the drivers of food insecurity, coping strategies, and associated mental health and sexual and reproductive health outcomes. By consolidating evidence across these interconnected domains, this review advances understanding of how food insecurity shapes the well-being of adolescent mothers and identifies critical research, policy, and programmatic gaps needed to inform interventions aimed at improving health outcomes and disrupting cycles of intergenerational disadvantage in SSA.

## Method

Our scoping review followed the framework of Arksey and O’Malley, which was further enhanced by the Joanna Briggs Institute [24]. The steps include (i) identifying the research question, (ii) identifying relevant studies, (iii) study selection, (iv) charting the data, and (v) collating, summarizing, and reporting the results [24]. We adhered to the Preferred Reporting Items for Systematic Reviews and Meta-Analyses extension for scoping reviews (PRISMA-ScR) guidelines (S1 Table) [25]. A systematic review was not considered suitable for our broad search scope, as it is primarily designed to address narrowly defined questions and test specific hypotheses [26,27]. A scoping review was deemed appropriate for identifying relevant literature, mapping available evidence, and identifying knowledge gaps [24]. In contrast to systematic reviews, formal methodological quality appraisal is not a required component of scoping review methodology [23]. Therefore, a methodological quality or risk of bias assessment was not conducted, as the primary objective of this review was to map the breadth and characteristics of the available evidence rather than evaluate the effectiveness of interventions or synthesize findings for causal inference.

### Step I. Identifying the research question

The Population, Concept and Context (PCC) mnemonic was used to guide the review, ensure clarity, and efficiently address the topic of the scoping review. P represents the population of adolescent mothers or pregnant adolescents aged 10–19 years, C represents the concept, which is “food insecurity,” and the context is “ sub-Saharan Africa”. Guided by the PCC framework and informed by RST, our scoping review addressed three key questions:

(1) What are the drivers of food insecurity among adolescent mothers (aged 10–19) in SSA?
(2) What coping strategies do adolescent mothers (aged 10–19) employ in response to food insecurity in SSA?
(3) What are the mental, sexual and reproductive health impacts of food insecurity among adolescent mothers (aged 10–19) in SSA?

### Step II. Identifying relevant studies

In consultation with University of Toronto librarians in May 2025, we conducted a literature search in Medline, Sociological Abstracts, Africa-Wide, PsycINFO, and Scopus. An updated search was conducted in February 2026.

Concepts guiding database searches were from three broad terms:

- food insecurity (including food scarcity, hunger, famine, malnutrition, undernutrition); AND
- Adolescent motherhood (young mothers, young motherhood, adolescent mom, teen moms, teenage mothers, teen pregnancy, teenage pregnancy)
- sub-Saharan Africa (Angola, Benin, Burundi, Burkina Faso, Botswana, Cameroon, Central African Republic, Chad, Cape Verde, Congo (Republic of the Congo, Democratic Republic of the Congo (DRC), Côte d’Ivoire Equatorial Guinea, Gabon, Sao Tome and Principe, Comoros, Djibouti, Eritrea, Ethiopia, Eswatini (formerly Swaziland), Gambia, Ghana, Guinea, Guinea-Bissau, Kenya, Lesotho, Liberia, Madagascar, Malawi, Mali, Mauritania, Mauritius, Mozambique, Namibia, Niger, Nigeria, Rwanda, Senegal, Seychelles, Sierra Leone, Somalia, South Africa, South Sudan, Tanzania, Togo, Uganda, Zambia, Zimbabwe)

#### Inclusion criteria.

All searches were restricted to English-language publications and studies focused on the SSA region. A 20-year search period (January 1, 2005, to January 1, 2025) was selected to ensure comprehensive coverage of contemporary evidence on food insecurity among pregnant and parenting adolescent mothers while capturing changes in the social, economic, and health contexts affecting adolescent mothers in SSA. An updated search was conducted from February 24 to 27, 2026, to identify new studies. Articles had to include adolescent mothers or pregnant adolescents aged between 10 and 19 years inclusive. We considered studies employing quantitative, qualitative, or mixed methods designs.

#### Exclusion criteria.

Articles were excluded if more than 50% of their participants fell outside the specified age range. Non-English publications were excluded from the review.

### Step III. Study selection

We first screened titles and abstracts of all identified articles independently. For records that passed this stage, we retrieved full-text articles and verified that they met the inclusion criteria. The process was conducted by two authors (SA & TI) using Covidence, a web-based platform designed for efficient systematic reviews [28]. Titles and abstracts were screened independently, with disagreements resolved through discussion; when consensus was not reached, the article advanced to full-text screening. Any disagreements at the full-text stage were resolved through consultation with a third author (KA). Reference lists of studies included in the full-text review were also searched to identify studies that met the inclusion criteria. Our search is provided in the S1 Appendix.

### Step IV. Data charting

Prior to conducting data extraction, we piloted the data extraction forms. Data extracted from each included study included author(s), year of publication, study title, journal/source, study design, population/participants, sample size, country, research objectives, key drivers, coping strategies, mental health impacts, sexual and reproductive health outcomes, and identified gaps. Two authors (SA & KA) participated in these pilot extractions. Each author independently extracted data from the first two sources using the draft form. After completing their extractions, they met to compare results, resolve discrepancies, and refine the data extraction form. This revised version was used for all subsequent extractions.

### Step V. Collating, summarizing, and reporting the results

The charted data were subsequently analyzed and synthesized in alignment with the review’s overarching aim and sub-questions. We first developed a descriptive understanding of the characteristics of the included studies, then identified the drivers, coping strategies, and impacts of food insecurity, noting age-related variations. The results were presented in both narrative and tabular formats to facilitate clarity and comparison. Table 1 provides an overview of each study’s characteristics, including setting and specific adolescent mother group (pregnant or mothering). Building on this synthesis, we developed a conceptual diagram informed by the RST framework to visually represent the findings and enhance understanding of research on food insecurity among adolescent mothers in SSA.

## Result

The search results are presented in a PRISMA-ScR flow diagram [25], shown in Fig 1. A total of 311 results were identified using the search criteria, and then 48 duplicates were eliminated. Thereafter, two reviewers screened 263 articles for eligibility, and 240 articles were excluded (Fig 1). A full text review was conducted for 23 studies, and an additional four studies were identified from citation searching. A total of 16 articles were eligible and included for data extraction (Table 2).

**Fig 1:**
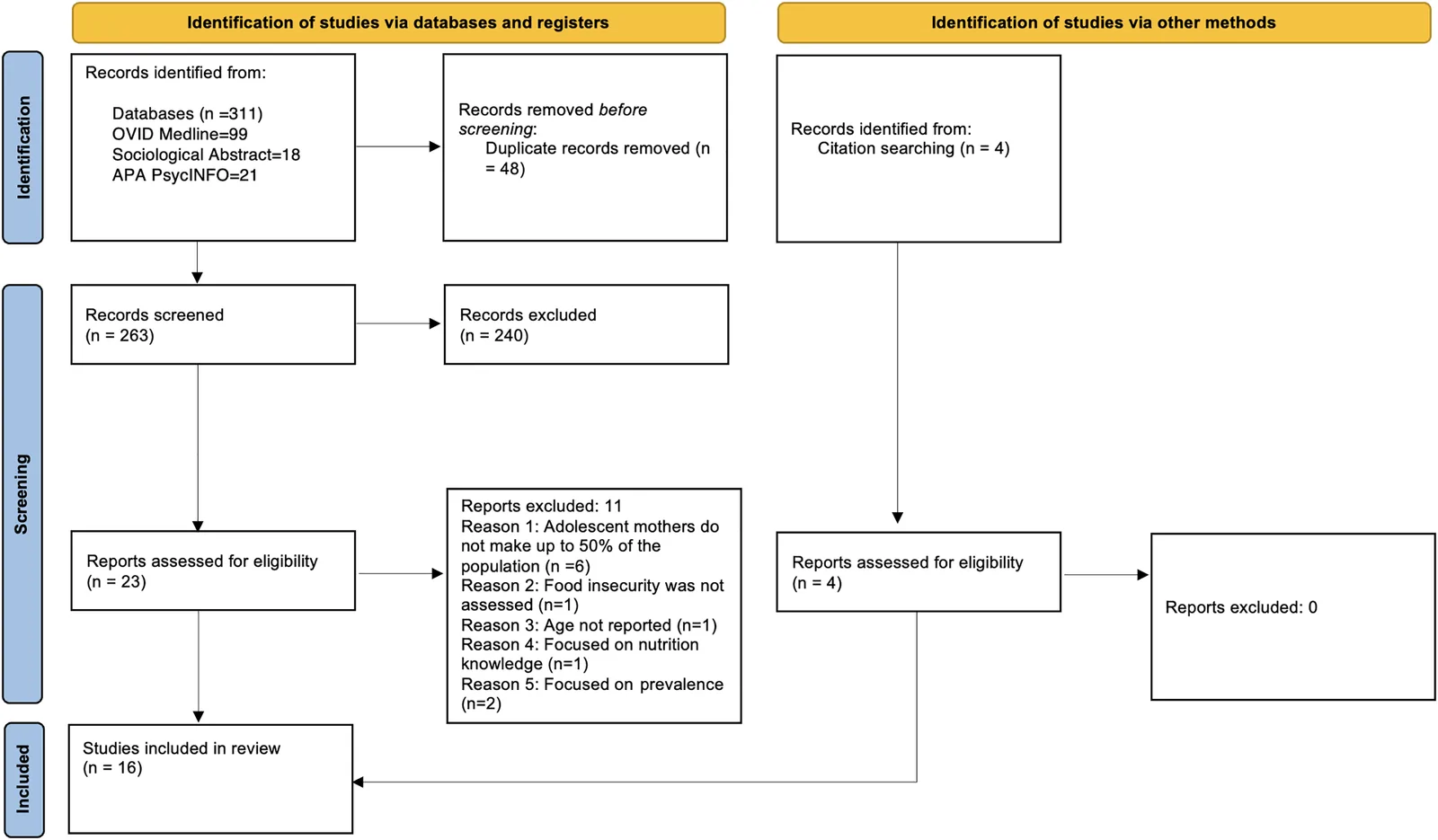
PRISMA flowchart of the number of unique records at each stage. Source: Page MJ, et al. BMJ 2021;372:n71. https://doi.org/10.1136/bmj.n71. This work is licensed under CC BY 4.0. To view a copy of this license, visit https://creativecommons.org/licenses/by/4.0/.

### General characteristics of included studies

The studies included in the review examined the food insecurity experiences of adolescent mothers in SSA countries (Table 1), using both qualitative and quantitative approaches. Most of the studies employed quantitative methods (n = 10). Ghana accounted for most studies (n = 7) [29–35], followed by South Africa [4,25,36–39], and Ethiopia [40,41]. Only single studies were identified from Nigeria [42], Malawi [43], and Uganda [44] (Fig 2). The articles are discussed below: categorized into an overview of drivers of food insecurity**,** coping strategies**,** and mental, sexual, and reproductive health (SRH) impacts. Five studies focused on pregnant adolescent mothers [29,30,40,42,43], and six studies examined parenting adolescent mothers [25–27,36,42,44] (Table 2). Three additional studies focused on both pregnant and parenting adolescent mothers [31–33]. While two studies by Carbone et al. (2019) and Toska et al. (2022) addressed adolescent mothers living with HIV.

**Table 1:** Summary of included study characteristics.

Author(s)	Year	Country	Study Design	Setting	Population (n)	Age (years)	Key findings
Ahmed et al.	2025	Dolo-Ado town, Somali region, Ethiopia	Quantitative	Community-based	Pregnant adolescents (n = 380)	15-19	Food insecurity was associated with undernutrition, while low household income was a significant predictor of undernutrition
Anima & Tampah-Naah	2023	Adaklu District of Ghana	Qualitative	Community-based	Adolescent mothers (n = 45)	13-19	Teenage mothers relied on farming and agricultural labour, alongside support from family, community members, and social networks, to secure food and livelihoods.
Boateng et al.	2023	Domeabra, a suburb of Winneba, the capital town of Effutu Municipality in the Central Region of Ghana	Qualitative	Community-based	Adolescent mothers (n = 40)	15-19	Poverty was identified as a key driver of food insecurity, with some adolescent mothers engaging in transactional sex to obtain food and meet basic household needs.
Carbone et al.	2019	Four Districts in Malawi (Mzimba North Region, Lilongwe, Thyolo, and Mangochi region)	Qualitative	Community-based	Adolescent mothers Living with HIV	15-19	Food shortages undermined ART adherence and well-being among adolescent mothers living with HIV.
Cluver et al.	2022	Two health municipalities of South Africa’s Eastern Cape Province	Quantitative	Health facility, school and community-based	Adolescent mothers (n = 1,024) (28% lived with HIV)	11-19	Food security was associated with lower odds of transactional sex, age-disparate sex, substance-related sex, and other HIV risk behaviours.
Cluver et al.	2025	East Cape of South Africa	Quantitative	Health facilities, secondary school, and community	Girls and young women who became mothers as adolescents	10-24	Food security was associated with lower odds of transactional or age-disparate sex, non-use of contraception, school or work disengagement, and poor self-efficacy
Doglikuu et al.	2023	20-electoral areas at Twifo-Atti-Morkwa district in the central region of Ghana	Quantitative	Community-based	Pregnant adolescents (n = 200)	10-20	Moderate and severe food insecurity were significantly associated with depression among pregnant adolescents.
Govender	2020	KwaZulu-Natal South Africa	Qualitative	Health facility based	Adolescent mothers (n = 18)	16-19	Financial hardship constrained food access.
Kassa et al.	2023	Northwest Ethiopia	Quantitative	Health facility based	Adolescent mothers (n = 422)	15-19	Household food insecurity was significantly associated with postpartum depression among adolescent mothers
Muwanguzi et al.	2025	Healthcare facilities in Mbarara City in South Western Uganda	Quantitative	Health facility based	Pregnant adolescents (n = 373)	13-19	Food insecurity was associated with more than twice the odds of depression among pregnant adolescents
Nsiah-Asamoah et al.	2024	Nine communities across 3 municipalities and 2 districts in the Central Region of Ghana.	Qualitative	Community-based	Pregnant adolescent and adolescent mothers (n = 35)	12-19	School dropout, limited literacy, inadequate income-generating skills, and lack of social support reduced livelihood opportunities and increased vulnerability to food insecurity among adolescent mothers.
Okronipa et al.	2025	Cape Coast in the Central Region of Ghana	Quantitative	Community-based	Pregnant adolescents (n = 216) & adolescent mothers (n = 206)	12-19	Food insecurity was highly prevalent (87%) and associated with poor dietary quality among pregnant adolescents and adolescent mothers.
Oladeji et al.	2022	30 selected primary maternal and child healthcare centers in Ibadan, Nigeria	Quantitative	Health facility based	Pregnant adolescents (n = 1,359)	14-20	Food insecurity was independently associated with being depressed among pregnant adolescents
Posey et al.	2024	Cape Coast, Ghana	Qualitative	Community-based	Pregnant adolescents & adolescent mothers (n = 34)	12-18	Poverty, stigma, infertile land, and poor infrastructure contributed to food insecurity; coping strategies included family support, informal work, transactional sex, and meal stretching.
Toska et al.	2022	Eastern Cape province of South Africa	Quantitative	Health facility based	Adolescent mothers (n = 311)	15-19	Food insecurity remained common among adolescent mothers, with lower child food security among those living with HIV despite access to food support services.
Quarshie et al.	2025	15 healthcare facilities across 16 administrative regions of Ghana	Quantitative	Health facility based	Pregnant adolescents (n = 449)	14-19	Food insecurity was significantly associated with suicidal ideation and suicide attempts among pregnant adolescents.

**Fig 2:**
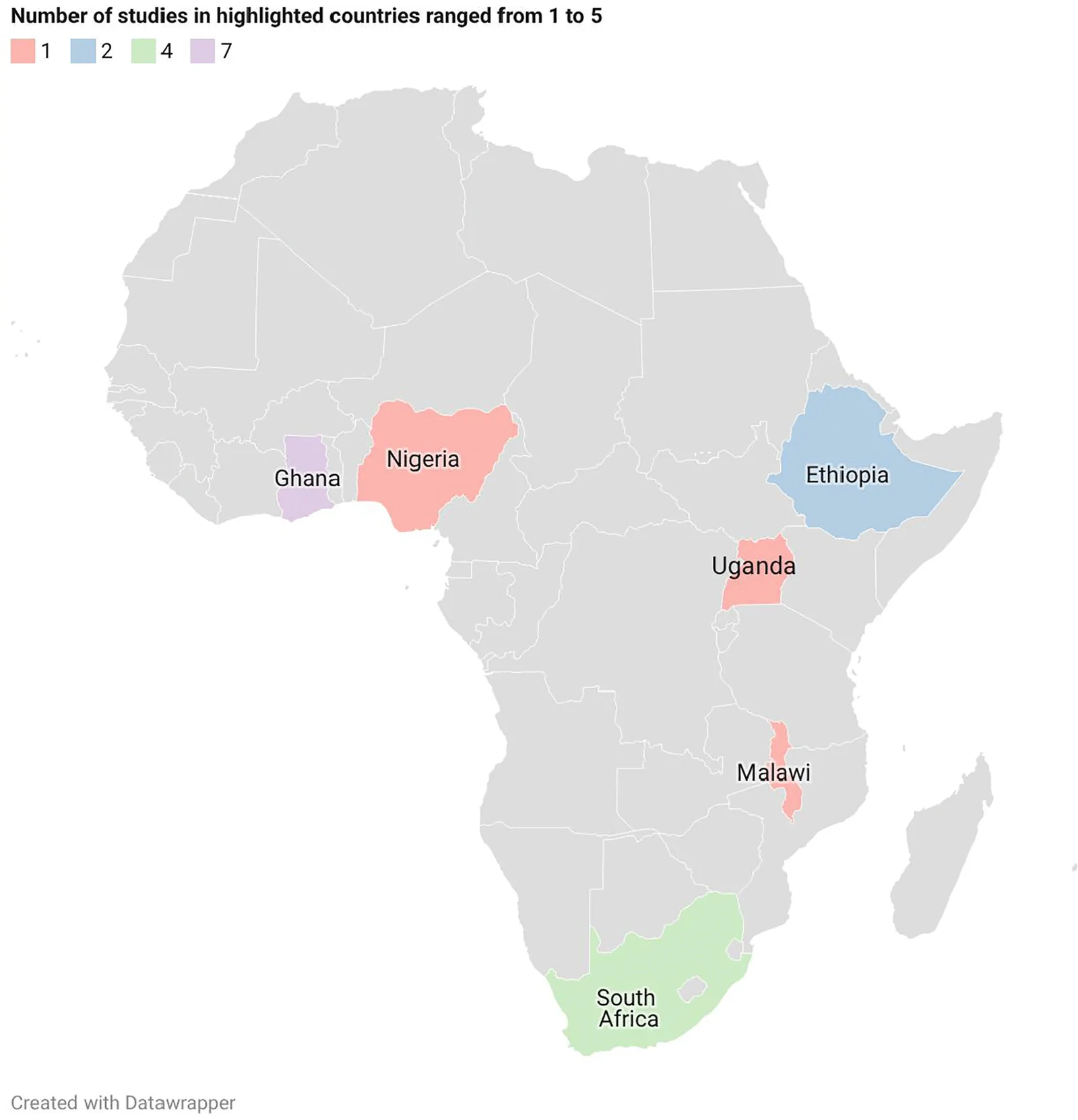
Geographical distribution of the 16 studies reviewed in this article.

Food insecurity was assessed using a range of measurement approaches across the included studies. Quantitative studies commonly employed validated instruments, including the Household Food Insecurity Access Scale (HFIAS) [29,40], the Child Food Insecurity Experience Scale (CFIES) [31], and measures derived from the South African National Food Consumption Survey [36]. One study used a locally validated household food insecurity questionnaire [45], while others used indicators such as food sufficiency [31], household hunger [30,42], or single-item measures of food insecurity [29,30]. Qualitative studies explored experiences of food insecurity through participant narratives. The heterogeneity in measurement approaches should be considered when comparing findings across studies.

### Drivers of food insecurity among adolescent mothers in SSA

Food insecurity among adolescent mothers was shaped by interlinked economic (e.g., poverty), social (e.g., stigma), and ecological (e.g., low agricultural productivity) factors.

#### Economic drivers.

Poverty and broader economic constraints consistently emerged as central drivers of food insecurity among adolescent mothers [35,37]. School dropout and limited employable or income-generating skills contributed to restricted livelihood opportunities, deepening financial vulnerability. In South Africa, adolescent mothers who were out of school experienced persistent economic hardship that limited their ability to secure adequate food [36]. Similarly, studies from Ghana found that school dropout was associated with limited literacy and a lack of income-generating skills among adolescent mothers, reducing opportunities for earning an income and increasing their risk of food insecurity and hunger [32,34].

Limited access to employment further reduced financial resources, reinforcing poverty as a key driver of food insecurity [31]. Additional evidence from Ghana identified poverty as the primary determinant of food insecurity among adolescent mothers [30,35]. Govender (2020) likewise reported that financial hardship directly affected the ability of adolescent mothers to feed themselves and their children.

Although focused on undernutrition rather than food insecurity, findings by Ahmed et al. (2025) also underscore the economic dimension of nutritional vulnerability. In Dolo-Ado town, on the eastern border of Ethiopia, which is a large refugee camp for Somali refugees,

low household income was significantly associated with undernutrition among pregnant adolescents (AOR = 2.6; 95% CI: 1.2–5.4), along with rural residence and low maternal education. While the outcome measured was undernutrition, the underlying driver, which is insufficient household income, is closely linked to the economic constraints identified across studies.

#### Social drivers.

Food insecurity among adolescent mothers was influenced by stigma, family size and caregiving responsibilities. Stigma surrounding adolescent pregnancy repeatedly emerged as a critical barrier [33–35,39]. Social stigma related to early pregnancy often resulted in rejection by family and community members, affecting access to food. For instance, in Ghana, Posey et al. (2024) revealed that stigma related to unwanted pregnancies, compounded by a lack of community infrastructure, significantly restricted adolescent mothers’ ability to secure adequate food. Family size and caregiving responsibilities also intensified food insecurity, particularly in resource-limited settings [31].

#### Ecological drivers.

Environmental constraints, such as infertile land and poor community infrastructure, further reduced food availability and access. In peri-urban communities in Cape Coast, Ghana, limited agricultural productivity due to degraded soils and a lack of irrigation restricted food production among adolescent mothers, with some areas reporting soil degradation linked to illegal mining activities [33]. Anima & Tampah-Naah (2023) found that in the Adaklu District of Ghana, school adolescent mothers often relied on on-farm activities as their main means of survival [34]. With few livelihood options and low patronage for non-farming ventures due to poor road conditions and distance from major cities, many engaged in planting or harvesting crops for others. In return for their labour, adolescent mothers sometimes received portions of the harvested produce, such as corn or beans, which became the primary food source for themselves and their children.

### Coping strategies

Coping strategies adopted by adolescent mothers in response to food insecurity can be broadly categorized as economic-based, socially oriented and survival responses shaped by structural vulnerability. Economic-based strategies included informal work, trading, borrowing food, pawning personal items, and meal stretching [33,34]. Social coping mechanisms involved reliance on family and community support, though this was inconsistent and often contingent on family relationships [37]. Migration to urban areas in search of better employment opportunities was also reported as a livelihood-enhancing strategy among adolescent mothers in Ghana [34].

Studies also documented survival responses linked to severe food insecurity, including transactional sex and coercive sexual relationships [33,35]. These experiences reflected broader structural vulnerabilities, including poverty, limited livelihood opportunities, social exclusion, and gendered power imbalances, which constrained adolescent mothers’ options for meeting their basic needs. Consistent with Resource Scarcity Theory, these findings demonstrate how resource scarcity can shape decision-making and increase exposure to sexual and reproductive health vulnerabilities.

### Outcomes

#### Mental health impacts.

Food insecurity had cascading effects on mental health, particularly depression and suicidal behaviour. Four studies reported significant associations between food insecurity and depression among adolescent mothers [29,42,44,45]. Doglikuu et al. (2023) found a significant association between food insecurity and depression among pregnant adolescent girls. Kassa et al. (2023) reported that postpartum depression risk was greater among food-insecure adolescent mothers compared to those who were food secure in Northwest Ethiopia. Evidence from Nigeria and Uganda also showed pregnant adolescents experiencing food insecurity had more than twice the odds of depression compared to those who were food secure [42,44]. Suicidal behaviour was also documented: in Ghana, food insecurity was associated with increased odds of suicide attempts among adolescent mothers (AOR =2.69; 95% CI 1.34 to 5.41) [30].

#### Sexual and reproductive health impacts.

Food insecurity contributed to a range of sexual, reproductive, and related physiological health vulnerabilities among adolescent mothers. Four studies documented sexual and reproductive health (SRH) risks linked to food insecurity [33,35,36,46]. In South Africa, Cluver et al. (2022) found that food insecurity was strongly associated with multiple HIV risk behaviours, including transactional sex (AOR = 0.17, 95% CI: 0.10–0.28), age-disparate sex, and sex under the influence of substances. The two qualitative studies by Posey et al. (2024) and Boateng et al. (2023) further illustrated how structural vulnerabilities, including poverty, constrained adolescent mothers’ options for meeting basic needs, including food, and increased their exposure to transactional sexual relationships and sexual coercion, thereby heightening SRH risks [33,35,36,46].

Food insecurity was also associated with physiological consequences that directly affect SRH outcomes. Evidence from Ahmed et al., (2025) and Zewdie et al. (2021) showed that household food insecurity was linked to undernutrition among adolescent mothers, a condition that heightens risks of anemia, obstetric complications, and poorer pregnancy outcomes. Carbone et al. (2019) further reported that food insecurity intensified the challenges of adhering to antiretroviral therapy, with adolescents describing physical weakness when taking medication without sufficient food and missing doses when food was unavailable. These findings highlight how inadequate food access not only increases SRH risk behaviours but also undermines the nutritional and treatment conditions necessary for healthy pregnancy and reproductive well-being.

## Discussion

Findings from this scoping review provide a comprehensive overview of the economic, social, and ecological drivers, coping strategies, mental health, and SRH impacts of food insecurity among adolescent mothers in SSA. The studies were sparsely distributed across the region, with most studies concentrated in Ghana, South Africa, and Ethiopia, reflecting geographic research gaps (Fig 2). These findings can inform future research, interventions, and policy addressing food insecurity among adolescent mothers in SSA.

Applying Resource Scarcity Theory (RST) provided a useful lens for understanding how social, economic, and ecological vulnerabilities intersect to shape food insecurity among adolescent mothers in SSA [15]. Across the included studies, structural drivers such as poverty, educational disruption, stigma, caregiving responsibilities, limited livelihood opportunities, and environmental constraints contributed to conditions of resource scarcity that constrained adolescent mothers’ ability to meet their basic needs. In response, adolescent mothers adopted a range of economic, social, and survival-oriented strategies to navigate food insecurity. These experiences were further associated with adverse mental, sexual, and reproductive health outcomes, including depression, suicidal ideation, HIV-related risk behaviours, and poor treatment adherence. Drawing on these interconnected findings, we developed a conceptual framework (Fig 3) that illustrates the pathways linking structural drivers, food insecurity, coping and survival responses, and health outcomes among adolescent mothers in SSA.

**Fig 3:**
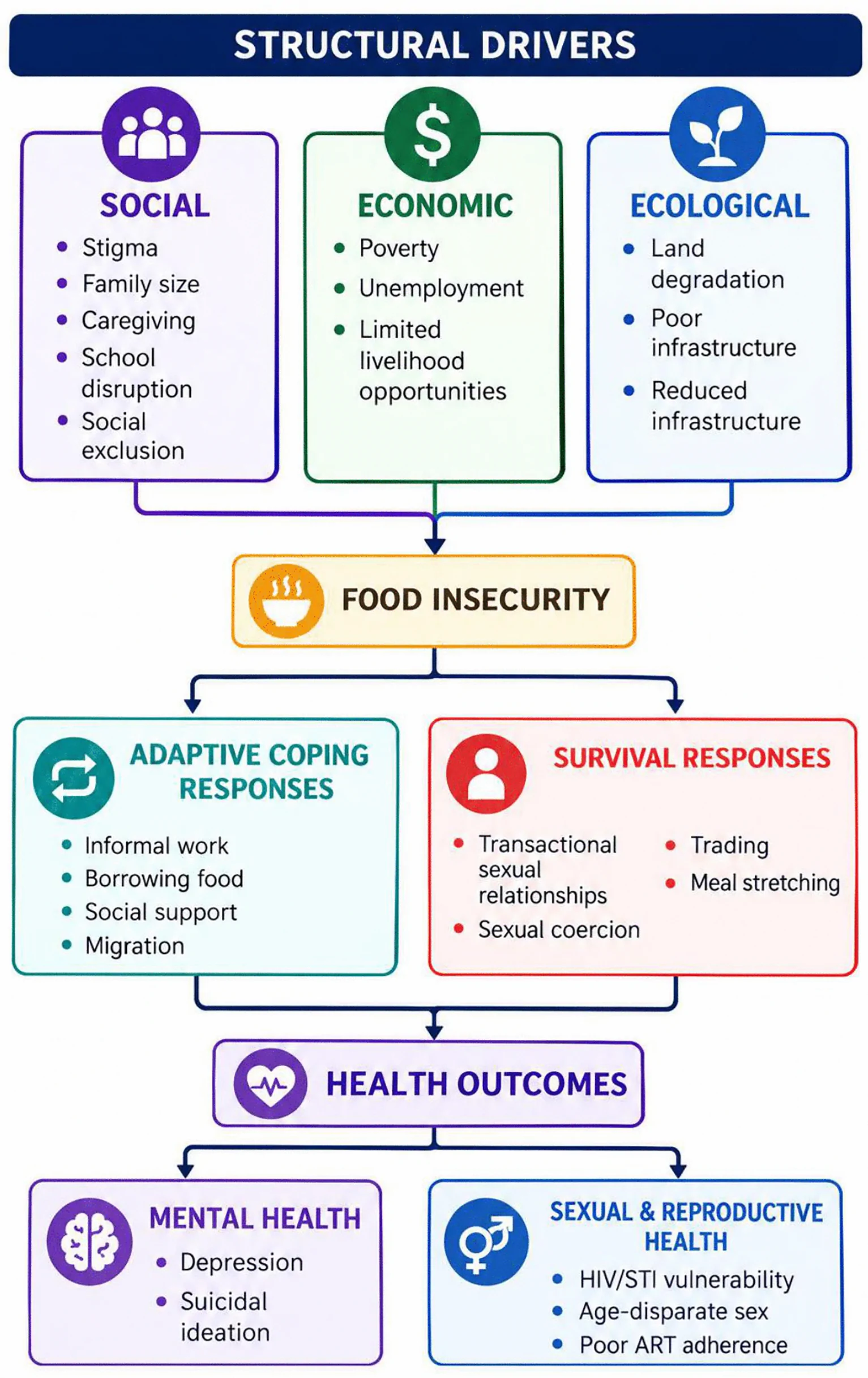
Conceptual framework of social, economic, and ecological drivers, coping responses, and health impacts of food insecurity among adolescent mothers in SSA.

Ecological drivers were particularly critical for adolescent mothers living in rural communities who depended on agriculture for their livelihoods. This factor is under-researched. Just one study in Cape Coast, Ghana, reported how anthropogenic environmental degradation rendered the soil infertile, highlighting systemic issues [33]. Although evidence from this review was limited, these findings should be understood within the broader context of environmental vulnerability in SSA, where declining agricultural productivity, land degradation, and climate-related shocks may reduce food availability and access among households dependent on agriculture [47]. Adolescent mothers may be especially vulnerable to these disruptions because of their limited economic resources and dependence on household and community food systems. Further research is needed to better understand how environmental and climate-related factors intersect with social and economic vulnerabilities to shape food insecurity among adolescent mothers in SSA.

Our review also noted how food insecurity was consistently linked to poor mental health outcomes. This pattern mirrors evidence from other low-resource settings in SSA and high-income countries where food insecurity amplifies the psychological burden [48,49]. For example, research among low-income single mothers, older than 18 years in Virginia, USA, showed that those experiencing food security reported poor mental health [48]. Similarly, findings among displaced and refugee youths in Kampala reported a similar association between chronic food insecurity and depression [49]. These findings align with evidence on the mental health consequences of uncertainty in obtaining food, feelings of shame, and awareness of social inequities [50]. Future studies should consider examining the pathways from food insecurity to poor mental health among adolescent mothers [50].

Food insecurity had significant implications for sexual and reproductive health among adolescent mothers. Limited access to food and economic resources due to broader structural inequalities increased vulnerability to transactional and coercive sexual relationships among some adolescent mothers [33]. These experiences should be understood within the context of constrained economic opportunities, social disadvantage, and gendered power relations, rather than as individual risk-taking behaviours. Evidence from South Africa further demonstrated that food security was associated with lower odds of transactional sex, age-disparate sex, and other HIV-related risk behaviours [36], suggesting that addressing food insecurity may reduce exposure to adverse sexual and reproductive health outcomes.

While the study by Toska et al. (2022) reported that food insecurity did not differ by HIV status, the rates of food security among children born to adolescent mothers living with HIV were lower compared to adolescent mothers who are HIV negative. This suggests that the effects of food insecurity may extend intergenerationally and intersect with maternal HIV status.

These findings highlight several areas for intervention and program development. For governments and social protection agencies, the evidence suggests a potential role for food assistance, cash transfer programs, and livelihood support initiatives targeted at pregnant and parenting adolescents [29,34]. Within the education sector, school re-entry initiatives, literacy programs, and vocational skills training may help address educational disruption and improve future economic opportunities [51]. Healthcare providers may benefit from integrating food insecurity screening and referral pathways into adolescent-friendly sexual and reproductive health, antenatal, postnatal, and mental health services [52]. Collectively, these approaches may help address the interconnected social, economic, and health challenges associated with food insecurity among adolescent mothers, although further intervention research is needed to determine their effectiveness.

Existing research has largely focused on links between food insecurity and HIV/STI vulnerability, with limited evidence on its effects on other critical dimensions of sexual health, such as sexual and gender-based violence (GBV), sexual function, and psychosexual counselling, and reproductive health, including antenatal, intrapartum, and postnatal care; contraception, fertility care, and safe abortion care [53]. Further research on other marginalized adolescent mothers, including those living with disabilities, was not available. Addressing these gaps is essential for developing holistic interventions that integrate food security into broader SRH service delivery for adolescent mothers.

Some research gaps were evident from this review. Existing studies are also geographically concentrated in a small number of SSA countries, with large regions remaining unrepresented, and rarely consider ecological drivers of food insecurity. While food insecurity is known to heighten SRH risks through pathways such as transactional or coerced sex and increased vulnerability to HIV and other STIs, its relationship to other sexual health domains, including sexual and gender-based violence, sexual function, psychosexual counselling, and comprehensive sexuality education, remains largely unexamined. Similar gaps exist for reproductive health areas such as antenatal, intrapartum, and postnatal care; contraception; fertility care; and safe abortion care. Addressing these evidence gaps through geographically diverse longitudinal research that provides insight into causal pathways between food insecurity and poor health outcomes (mental health and SRH) in SSA will be critical for developing effective, context-specific interventions that address the intertwined challenges. Lastly, further investigation is needed to understand the role of stakeholders in supporting livelihood opportunities for adolescent mothers.

### Strengths and limitations

This review is among the first to comprehensively synthesize evidence on food insecurity among adolescent mothers in SSA through the lens of Resource Scarcity Theory (RST), encompassing social, economic, and ecological drivers, as well as health (mental, sexual and reproductive health) impacts. We ensured broad coverage of peer-reviewed literature, and the inclusion of both quantitative and qualitative studies allowed us to capture diverse perspectives. Adherence to PRISMA-ScR guidelines further strengthened transparency and reproducibility.

First, the search was restricted to English-language publications, which may have resulted in the exclusion of relevant studies published in other languages, particularly from Francophone and Lusophone countries in SSA. Consequently, the review may be subject to language bias and may not fully capture the breadth of evidence on food insecurity among adolescent mothers across the region. Second, because the review primarily included published literature, publication bias may have occurred if studies with non-significant or null findings were less likely to be published. Third, food insecurity was measured using a range of approaches across studies, including validated instruments, dietary indicators, and single-item measures of hunger and food sufficiency. This heterogeneity in definitions and measurement may limit comparability across studies and should be considered when interpreting the findings. Fourth, consistent with scoping review methodology, we did not undertake a formal methodological quality or risk of bias assessment of the included studies. Consequently, the strength and robustness of the available evidence may vary across studies, and the findings should be interpreted as a mapping of the existing evidence base rather than an evaluation of the quality or certainty of the evidence. Finally, the relatively small number of included studies and their concentration in a limited number of countries, particularly Ghana and South Africa, may affect the generalizability of the findings across the diverse contexts of SSA.

In addition, most quantitative studies included in this review employed cross-sectional designs, limiting the ability to establish temporal or causal relationships between food insecurity and observed mental health, sexual and reproductive health outcomes. Consequently, the associations identified should be interpreted with caution, and future longitudinal and intervention studies are needed to better understand the mechanisms through which food insecurity influences the well-being of adolescent mothers in SSA.

## Conclusion

Our scoping review highlights that food insecurity is a critical yet underexamined determinant of adolescent mothers’ health and well-being in Sub-Saharan Africa. Applying the Resource Scarcity Theory highlighted how resource scarcity shapes the health and well-being of adolescent mothers in SSA. Future research should move beyond predominantly cross-sectional, quantitative designs to include longitudinal, qualitative, and mixed methods approaches that capture lived experiences, relational dynamics, and causal pathways. Expanding the scope of inquiry to underexplored SRH domains will be vital to advancing holistic understandings of adolescent mothers’ needs. By centring adolescent mothers in future research and policy, stakeholders can ensure that solutions are not only evidence-informed but also sustainable, equitable, and transformative.

## Supporting information

S1 Table Preferred Reporting Items for Systematic reviews and Meta-Analyses extension for Scoping Reviews (PRISMA-ScR) Checklist. (PDF)

S1 Appendix Overview of search strategy. (PDF)
