# Supplementary material for: Food insecurity among adolescent girls who are mothers (10–19 years) in sub-Saharan Africa: A scoping review
Source: PLoS One. 2026 Sep 30;21(9):e0346835. doi: 10.1371/journal.pone.0346835 (PMC13626366; doi:10.1371/journal.pone.0346835)
Supplement: Supplementary file 2 [file pone.0346835.s002.pdf]

S1 Appendix. Overview of search strategy

Sociological Abstract (27th of February 2026)

(SU("Food security") OR SU("Food supply") OR SU("Nutrition") OR SU("Malnutrition") OR MAINSUBJECT.EXACT("Food Insecurity") OR MAINSUBJECT.EXACT("Malnutrition") OR TIAB((food NEAR/3 (insecure* OR secur* OR availab* OR affordab* OR shortage OR depriv*))) OR TIAB(famine OR "food deserts" OR "access to healthy foods" OR hunger OR starvation OR "food scarcity") OR TIAB(malnutrition OR undernutrition OR malnourish* OR starvation OR hunger OR "food scarcity") OR TIAB(nutrition* NEAR/3 (deficien* OR adequat* OR essential)))AND(SU("Adolescent mothers") OR SU("Teenage pregnancy") OR SU("Adolescence") OR MAINSUBJECT.EXACT("Pregnancy in Adolescence") OR TIAB(adolescent mother* OR adolescent pregnancy OR "teen mom*" OR "teen mother*" OR "early motherhood") OR TIAB(pregnan* NEAR/3 (adolescen* OR teen* OR preteen*)))AND(SU("Africa South of the Sahara") OR SU("Sub-Saharan Africa") OR TIAB("Africa south of the Sahara" OR "Sub-Saharan Africa" OR Cameroon OR "Central African Republic" OR Chad OR Congo OR "Democratic Republic of the Congo" OR Gabon OR Rwanda OR Ethiopia OR Kenya OR Uganda OR Tanzania OR Sudan OR "South Sudan" OR Somalia OR Angola OR Malawi OR Mozambique OR Namibia OR "South Africa" OR Zambia OR Zimbabwe OR Benin OR Ghana OR Nigeria OR Senegal OR Togo OR Mali OR Niger OR Liberia OR "Sierra Leone" OR Guinea))

PsycINFO

OVID Medline

Ovid MEDLINE(R) February 16, 2026

#	Search Statement	Results	Annotation
1	exp Food Insecurity/	3120	
2	exp Food security/	1377	
3	(food adj3 (insecur* or secur*)).tw,kf.	24394	
4	(famine or food deserts or or access to healthy foods).tw,kf.	32610	
5	(hunger or malnutrition or undernutrition or malnourish* or famine or food scarcity or starvation).tw,kf.	153517	
6	(food adj3 (availab* or affordab* or deprived* or short*)).tw,kf.	16717	
7	(nutrition* adj3 (deficien* or adequat*or essential)).tw,kf.	9499	
8	exp malnutrition/	143787	
9	Access to Healthy Foods/	39	
10	food deserts/	57	
11	Famine/	278	
12	1 or 2 or 3 or 4 or 5 or 6 or 7 or 8 or 9 or 10 or 11	285550	
13	Adolescent Mothers/	104	
14	Pregnancy in Adolescence/	8957	
15	(teen* mom or teen* mother* or pregnancy in adolescence or early motherhood).tw,kf.	6492	

16	(pregnan* adj3 (adolescen* or teen* or preteen*)).tw,kf.	9840	
17	13 or 14 or 15 or 16	14413	
18	exp "Africa South of the Sahara"/	294083	
19	(africa south of the sahara or africa, central or cameroon or central african republic or chad or congo or democratic republic of the congo or equatorial guinea or gabon or rwanda or sao tome and principe or africa, eastern or burund or comoros or djibouti or eritrea or ethiopia or kenya or madagascar or seychelles or somalia or south sudan or sudan or tanzania or uganda or africa, southern or angola or botswana or eswatini or lesotho or malawi or mozambique or namibia or south africa or zambia or zimbabwe or africa, western or benin or burkina faso or cabo verde or cote d'ivoire or gambia or ghana or guinea or guinea-bissau or liberia or mali or mauritania or niger or nigeria or senegal or sierra leone or togo).tw,kf.	426625	
20	18 or 19	519366	
21	12 and 17 and 20	99	

APA PSYCH INFO (26th February, 2026)

#	**Query**	**Results from 24 Feb 2026**
1	exp food Insecurity/	2,186
2	exp hunger/	2,309
3	exp food deprivation/	3,575
4	exp starvation/	473
5	(food adj3 (insecure* or secur*)).tw.	4330
6	(hunger or malnutrition or food depriv* or undernutrition or malnourish* or famine or food scarcity or starvation).tw.	17933
7	(food adj3 (availab* or afford* or deprived* or short*)).tw.	5,553
8	(nutrition* adj3 (deficien* or adequat*or essential)).tw.	715
9	exp malnutrition/	36,643
10	(food deserts).tw.	96
11	1 or 2 or 3 or 4 or 5 or 6 or 7 or 8 or 9 or 10	59,485
12	exp adolescent mothers/	2,656
13	exp adolescent pregnancy/	3,281

14	(Adolescent mother* or adolescent pregnancy or teen* mom or teen* mother* or pregnancy in adolescence or early motherhood).tw.	4,392
15	(pregnan* adj3 (adolescen* or teen* or preteen*)).tw.	5,048
16	12 or 13 or 14 or 15	8,341

17	("africa south of the sahara" or africa, central or cameroon or central african republic or chad or congo or "democratic republic of the congo" or equatorial guinea or gabon or rwanda or "sao tome and principe" or africa, eastern or burund or comoros or djibouti or eritrea or ethiopia or kenya or madagascar or seychelles or somalia or south sudan or sudan or tanzania or uganda or africa, southern or angola or mauritius or botswana or eswatini or lesotho or malawi or mozambique or namibia or south africa or zambia or zimbabwe or africa, western or benin or burkina faso or cabo verde or cote d'ivoire or gambia or ghana or guinea or guinea-bissau or liberia or mali or mauritania or niger or nigeria or senegal or sierra leone or togo).tw.	59,753
18	11 and 16 and 17	21

Scopus (26TH February, 2026) (TITLE-ABS-KEY("food insecur*" OR "food secur*" OR "food insufficien*" OR "food scarcity" OR hunger OR famine OR starvation OR malnutrition

OR undernutrition OR malnourish* OR (food W/3 (insecure OR insecurity OR secure OR security)) OR (food W/3 (availab* OR affordable OR affordability OR deprived* OR short* OR shortage*)) OR

(nutrition* W/3 (deficien* OR inadequat* OR adequat* OR essential)) OR "food desert*" OR "access to healthy food*" OR "access to food") OR INDEXTERMS("food insecurity" OR "food security" OR hunger OR famine OR malnutrition OR "food supply" OR poverty))AND(TITLE-ABS-KEY("adolescent mother*" OR "adolescent pregnancy" OR "pregnancy in adolescence" OR "teen* mother*" OR "teen* mom*" OR "teenage mother*" OR "young mother*" OR "early motherhood" OR (pregnan* W/3 (adolescen* OR teen* OR preteen* OR youth*)) OR (childbearing W/3 adolescen*)) OR INDEXTERMS("adolescent pregnancy" OR "adolescent mothers"))AND(TITLE-ABS-KEY("sub-Saharan Africa" OR "Africa south of the Sahara" OR "central Africa" OR "eastern Africa" OR "southern Africa" OR "western Africa" OR Angola OR Benin OR Botswana OR "Burkina Faso" OR Burundi OR Cameroon OR "Cape Verde" OR "Central African Republic" OR Chad OR Comoros OR Congo OR "Republic of the Congo" OR "Democratic Republic of the Congo" OR "Côte d'Ivoire" OR Djibouti OR Eritrea OR Eswatini OR Ethiopia OR Gabon OR Gambia OR Ghana OR Guinea OR "Guinea-Bissau" OR Kenya OR Lesotho OR Liberia OR Madagascar OR Malawi OR Mali OR Mauritania OR Mozambique OR Namibia OR Niger OR Nigeria OR Rwanda OR Senegal OR Seychelles OR Sierra Leone OR Somalia OR "South Africa" OR "South Sudan" OR Tanzania OR Togo OR Uganda OR Zambia OR Zimbabwe))

Africa-wide (24TH February, 2026)

(TI ("food insecure*" OR "food secur*" OR (food N3 (insecure* OR secure*)) OR famine

OR "food desert*" OR "access to healthy food" OR hunger OR malnutrition OR undernutrition OR undernourish* OR malnourish* OR "food scarcity" OR starvation OR

(food N3 (availability OR available OR affordab* OR depriv* OR shortage)) OR (nutrition* N3 (deficien* OR adequate* OR essential))) OR AB ("food insecure*" OR "food secur*" OR (food N3 (insecure* OR secure*)) OR famine OR "food desert*" OR "access to healthy food" * OR hunger OR malnutrition OR undernutrition OR undernourish* OR malnourish* OR "food scarcity" OR starvation OR (food N3 (availab* OR afford* OR depriv* OR shortage)) OR (nutrition* N3 (deficien* OR adequate* OR essential))) OR KW ("food insecure*" OR "food secur*" OR (food N3 (insecure OR security)) OR famine OR "food desert*" OR "access to healthy food" *OR hunger OR malnutrition OR undernutrition OR undernourish* OR malnourish* OR "food scarcity" OR starvation OR (food AND (availab* OR affordab* OR depriv* OR shortage)) OR (nutrition* N3 (deficien* OR adequate* OR essential)))) AND (TI ("adolescent mother*" OR "adolescent pregnancy" OR "pregnancy in adolescence" OR "teen* mother*" OR "teen* mom*" OR "early motherhood" OR (pregnan* AND (adolescen* OR teen* OR preteen*))) OR AB ("adolescent mother*" OR "adolescent pregnancy" OR "pregnancy in adolescence" OR "teen* mother*" OR "teen* mom*" OR "early motherhood" OR (pregnan* AND (adolescen* OR teen* OR preteen*))) OR KW ("adolescent mother*" OR "adolescent pregnancy" OR "pregnancy in adolescence" OR "teen* mother*" OR "teen* mom*" OR "early motherhood" OR (pregnan* AND (adolescen* OR teen* OR preteen*)))) AND (TI ("sub-saharan africa" OR "africa south of the sahara" OR "central africa" OR cameroon OR "central african republic" OR chad OR congo OR "democratic republic of the congo" OR "republic of the congo" OR "equatorial guinea" OR gabon OR rwanda OR "sao tome and principe" OR "eastern africa" OR burundi OR comoros OR djibouti OR eritrea OR ethiopia OR kenya OR madagascar OR seychelles OR somalia OR "south sudan" OR sudan OR tanzania OR uganda OR "southern africa" OR angola OR botswana OR eswatini OR lesotho OR malawi OR mozambique OR namibia OR "south africa" OR zambia OR zimbabwe OR "western africa" OR benin OR "burkina faso"

OR "cabo verde" OR "cote d'ivoire" OR gambia OR ghana OR guinea OR "guinea-bissau" OR liberia OR mali OR mauritania OR niger OR nigeria OR senegal OR "sierra leone" OR togo) OR AB ("sub-saharan africa" OR "africa south of the sahara" OR "central africa" OR cameroon OR "central african republic" OR chad OR congo OR "democratic republic of the congo" OR "republic of the congo" OR "equatorial guinea" OR gabon OR rwanda OR "sao tome and principe" OR "eastern africa" OR burundi OR comoros OR djibouti OR eritrea OR ethiopia OR kenya OR madagascar OR seychelles OR somalia OR "south sudan" OR sudan OR tanzania OR uganda OR "southern africa" OR angola OR botswana OR eswatini OR lesotho OR malawi OR mozambique OR namibia OR "south africa" OR zambia OR zimbabwe OR "western africa" OR benin OR "burkina faso" OR "cabo verde" OR "cote d'ivoire" OR gambia OR ghana OR guinea OR "guinea-bissau" OR liberia OR mali OR mauritania OR niger OR nigeria OR senegal OR "sierra leone" OR togo) OR KW ("subsaharan africa" OR "africa south of the sahara" OR "central africa" OR cameroon OR "central african republic" OR chad OR congo OR "democratic republic of the congo" OR "republic of the congo" OR "equatorial guinea" OR gabon OR rwanda OR "sao tome and principe" OR "eastern africa" OR burundi OR comoros OR djibouti OR eritrea OR ethiopia OR kenya OR madagascar OR seychelles OR somalia OR "south sudan" OR sudan OR tanzania OR uganda OR "southern africa" OR angola OR botswana OR eswatini OR lesotho OR malawi OR mozambique OR namibia OR "south africa" OR zambia OR zimbabwe OR "western africa" OR benin OR "burkina faso" OR "cabo verde" OR "cote d'ivoire" OR gambia OR ghana OR guinea OR "guinea-bissau" OR liberia OR mali OR mauritania OR niger OR nigeria OR senegal OR "sierra leone" OR togo))
